# Chronic Hypoxia Promotes Pulmonary Artery Endothelial Cell Proliferation through H_2_O_2_-Induced 5-Lipoxygenase

**DOI:** 10.1371/journal.pone.0098532

**Published:** 2014-06-06

**Authors:** Kristi M. Porter, Bum-Yong Kang, Sherry E. Adesina, Tamara C. Murphy, C. Michael Hart, Roy L. Sutliff

**Affiliations:** Emory University School of Medicine/Atlanta Veterans Affairs Medical Center, Department of Pulmonary, Allergy and Critical Care Medicine, Atlanta, Georgia, United States of America; University of Pittsburgh School of Medicine, United States of America

## Abstract

Pulmonary Hypertension (PH) is a progressive disorder characterized by endothelial dysfunction and proliferation. Hypoxia induces PH by increasing vascular remodeling. A potential mediator in hypoxia-induced PH development is arachidonate 5-Lipoxygenase (ALOX5). While ALOX5 metabolites have been shown to promote pulmonary vasoconstriction and endothelial cell proliferation, the contribution of ALOX5 to hypoxia-induced proliferation remains unknown. We hypothesize that hypoxia exposure stimulates HPAEC proliferation by increasing ALOX5 expression and activity. To test this, human pulmonary artery endothelial cells (HPAEC) were cultured under normoxic (21% O_2_) or hypoxic (1% O_2_) conditions for 24-, 48-, or 72 hours. In a subset of cells, the ALOX5 inhibitor, zileuton, or the 5-lipoxygenase activating protein inhibitor, MK-886, was administered during hypoxia exposure. ALOX5 expression was measured by qRT-PCR and western blot and HPAEC proliferation was assessed. Our results demonstrate that 24 and 48 hours of hypoxia exposure have no effect on HPAEC proliferation or ALOX5 expression. Seventy two hours of hypoxia significantly increases HPAEC ALOX5 expression, hydrogen peroxide (H_2_O_2_) release, and HPAEC proliferation. We also demonstrate that targeted ALOX5 gene silencing or inhibition of the ALOX5 pathway by pharmacological blockade attenuates hypoxia-induced HPAEC proliferation. Furthermore, our findings indicate that hypoxia-induced increases in cell proliferation and ALOX5 expression are dependent on H_2_O_2_ production, as administration of the antioxidant PEG-catalase blocks these effects and addition of H_2_O_2_ to HPAEC promotes proliferation. Overall, these studies indicate that hypoxia exposure induces HPAEC proliferation by activating the ALOX5 pathway via the generation of H_2_O_2_.

## Introduction

Pulmonary Hypertension (PH) is a progressive disorder characterized by sustained increases in pulmonary arterial pressures and vascular remodeling. Although the mechanisms underlying PH remain unknown, hypoxia induces PH in experimental models and is believed to contribute to disease development [1], [2] by impairing endothelial cell function [3], [4] as evidenced by abnormal endothelial cell growth in lung sections and pulmonary artery endothelial cells from PH patients [5], [6]. Altered expression of arachidonate 5-lipoxygenase (ALOX5), the enzyme that catalyzes the production of vasoactive leukotrienes from arachidonic acid, is associated with endothelial proliferation and PH development. Previous studies demonstrate that patients with idiopathic pulmonary hypertension exhibit increased ALOX5 expression in lung tissue, particularly in small pulmonary artery endothelial cells. Also, inhibition of ALOX5 or its required cofactor, 5-lipoxygenase activating protein (FLAP) attenuates hypoxia- or monocrotaline (MCT)-induced PH [7], [8], whereas ALOX5 overexpression accelerates and exacerbates PH in MCT-treated rats [9]. ALOX5 metabolites, such as the cysteinyl leukotrienes (CysLT) are suggested to mediate these effects as they induce vasoconstriction in the distal segments of pulmonary arteries [10]. Moreover, inhibitors of CysLT production attenuate proliferation of pulmonary artery endothelial cells [11].

ALOX5 activity and leukotriene production are regulated by numerous signaling pathways. Primarily, ALOX5 requires the presence of FLAP for leukotriene synthesis [12], [13]. Yet, ALOX5 is also activated in conditions that promote lipid peroxidation [14] particularly following glutathione depletion [15], [16]. Studies also indicate that increases in endogenous reactive oxygen species (ROS) release stimulate ALOX5 expression [14] and cause an almost 4-fold increase in leukotriene formation [17]. These studies strongly suggest that ROS may induce ALOX5 expression. However, the connection between hypoxia-induced PH, ROS and endothelial ALOX5 is not completely understood. In this study, we investigate whether chronic hypoxia exposure alters endothelial ALOX5 expression, the effects of hypoxia-induced ALOX5 expression on endothelial cell proliferation and the role of hypoxia-induced ROS.

## Materials and Methods

### Reagents

Trypan blue, fetal bovine serum (FBS), dimethyl sulfoxide (DMSO), PEG-Catalase, and gelatin were obtained from Sigma-Aldrich (St. Louis, MO). Zileuton was obtained from Patheon Pharmaceuticals (Cincinnatti, OH). MK-886 was purchased from Calbiochem (San Diego, CA). Scrambled and silencing RNA (siRNA) for ALOX5 was obtained from Qiagen (Valencia, CA).

### Cell Culture

Human pulmonary artery endothelial cells (HPAEC) were obtained from Lonza Clonetics (Walkersville, MD). HPAEC were grown in EGM-2 medium (Lonza), which contains basic growth medium (EBM-2), fetal bovine serum (FBS), and antibiotics, ascorbic acid, vascular endothelial growth factor (VEGF), human fibroblast growth factor (hFGF-B), hydrocortisone, human epidermal growth factor (hEGF), R^3^-IGF-1 (insulin-like growth factor), GA-1000 (gentamicin, amphotericin B), and heparin. Unless otherwise stated, cells were maintained in a 37°C incubator at 5% CO_2_.

### Hypoxia Exposure

HPAEC, passages 3-8, were exposed to hypoxia in a Biospherix exposure chamber (Lacona, NY) as previously described [18], [19]. For normoxic conditions, HPAEC were placed into a standard incubator maintained at 37°C and 5% CO_2_ levels. For hypoxic conditions, HPAEC were placed in a hypoxia chamber maintained at 37°C, 1% oxygen, and 5% CO_2_ levels. Human pulmonary artery endothelial cells (HPAEC) were cultured in normoxic or hypoxic conditions for 24-, 48-, or 72 hours. To investigate the contribution of ROS levels, PEG-Catalase (1,000 U/mL) was administered during the final 24 hours of the 72 hours of hypoxia exposure. The role of 5-Lipoxygenase in HPAEC proliferation was studied by exposing cells to 10 µM concentrations of Zileuton or MK-886 either throughout the entire exposure period or during the final 24 hours.

### ALOX5 and FLAP mRNA Analysis

ALOX5 and FLAP mRNA levels were determined by quantitative real-time PCR (qRT-PCR) using the iCycler system (Bio-Rad, Hercules, CA). Total RNA was extracted from HPAEC using RNA-Bee. RNA concentrations were measured using the ND-1000 Spectrophotometer (NanoDrop Technology, Wilmington, DE). RNA (1.5 micrograms) was combined with random nanomer primers (Ambion), dNTPs (New England Bio-Labs) and nuclease-free water for reverse transcription. cDNA templates were amplified with gene-specific primer sets. All transcripts were detected using SYBR Green I (Molecular Probes, Inc). Transcripts were normalized to the housekeeping gene, β-Globin. Values are expressed as percent of control. Expression changes were determined using the 2^−ΔΔCt^ method.

### RNA Interference and HPAEC Transfection

Human ALOX5 siRNA (NM_000698), siRNA duplexes (5′- GGCAGGAAGACCTGATGTT -3′, target region 333–351) were designed using BLOCK-it RNAi Designer (Invitrogen). siRNA targeted to a specific noncoding gene was employed as a scrambled RNA control. At 40–50% confluence, HPAECs were transfected with scrambled or ALOX5 siRNA using GeneSilencer (Genlantis, San Diego, CA) transfection reagent according to manufacturer's instructions. After transfection for 6 hours, the transfection media was replaced with EGM containing 10% FBS. HPAECs were exposed to normoxia (NOR, 21% O_2_) or hypoxia (HYP, 1% O_2_) for 72 h. HPAEC lysates were then harvested and examined for ALOX5 levels using qRT-PCR and Western blots. In selected studies, HPAEC proliferation was determined using MTT assays.

### Western Blot Analyses

Following normoxia or hypoxia exposure, HPAEC lysates were subjected to Western Blot analysis as reported [18]. Primary antibodies for ALOX5 and GAPDH were purchased from Cayman Chemical Company (Ann Arbor, Michigan) and Sigma-Aldrich respectively. Proteins were visualized using fluorescent anti-goat or anti-rabbit secondary antibodies using the Licor system. Bands for protein of interest were quantified by densitometry and normalized to GAPDH levels within the same lane.

### Cell Proliferation Assay

Cell proliferation was assessed using the MTT (ATCC, Manassas, VA) Cell Proliferation Assay. Briefly, proliferating cells reduce the tetrazolium MTT resulting in intracellular formazan. Detergent reagent was added to cell to solubilize formazan. Supernatants were then collected and quantified using a spectrophotometer at 562 nm. Cell proliferation was further confirmed by cell counting using a trypan blue dye exclusion assay. Briefly, following hypoxia exposure, cells were trysinized and resuspended in medium. Cells were mixed with PBS and trypan blue at a 1∶8∶1 ratio. The trypan blue-negative cells were counted using a hemacytometer.

### Hydrogen Peroxide Analysis

Hydrogen Peroxide (H_2_O_2_) release was quantified using the Amplex Red Assay. Cells were incubated in a solution containing the Amplex Red reagent (Molecular Probes), horseradish peroxidase and a buffer solution for 30 minutes at 37°C. Supernatants were then collected and fluorescence measured at 560 nm. H_2_O_2_ concentrations were determined through standard curve extrapolation normalized to cellular protein concentration. Overall reactive oxygen species (ROS) and reactive nitrogen species were detected using the ROS-sensitive fluorescent probe 2′, 7′-dihydrodichlorofluorescein diacetate (DCF-DA; Invitrogen, Carlsbad, CA). Confluent HPAEC monolayers were loaded with 25 µg/mL DCF-DA for 1 hour at 37°C in Krebs–Ringer Phosphate Buffer (KRPG; 145 mM NaCl, 5.7 mM KH_2_PO_4_, 4.86 mM KCl, 0.54 mM CaCl_2_, 1.22 mM MgSO_4_, and 5.5 mM glucose, pH 7.35). A laser-scanning confocal microscope (Olympus, Center Valley, PA) and fluorimeter were used to detect DCF fluorescence at excitation and emission wavelengths of 488 nm and 520 nm, respectively.

### Statistical Analysis

A student's t-test analysis was used for comparison of two groups. One-way ANOVA with Tukey's posttest was used for the comparison of multiple groups. All experiments using cell cultures were repeated at least twice on different cell lines, and samples were run in duplicate or triplicate. Statistical significance was defined as P< 0.05, and all graphs are expressed as mean ± SEM. All statistical analyses were performed using GraphPad Prism software (La Jolla, CA).

## Results

### Prolonged Hypoxia Exposure Promotes Endothelial Cell Proliferation

**Figure 1:**
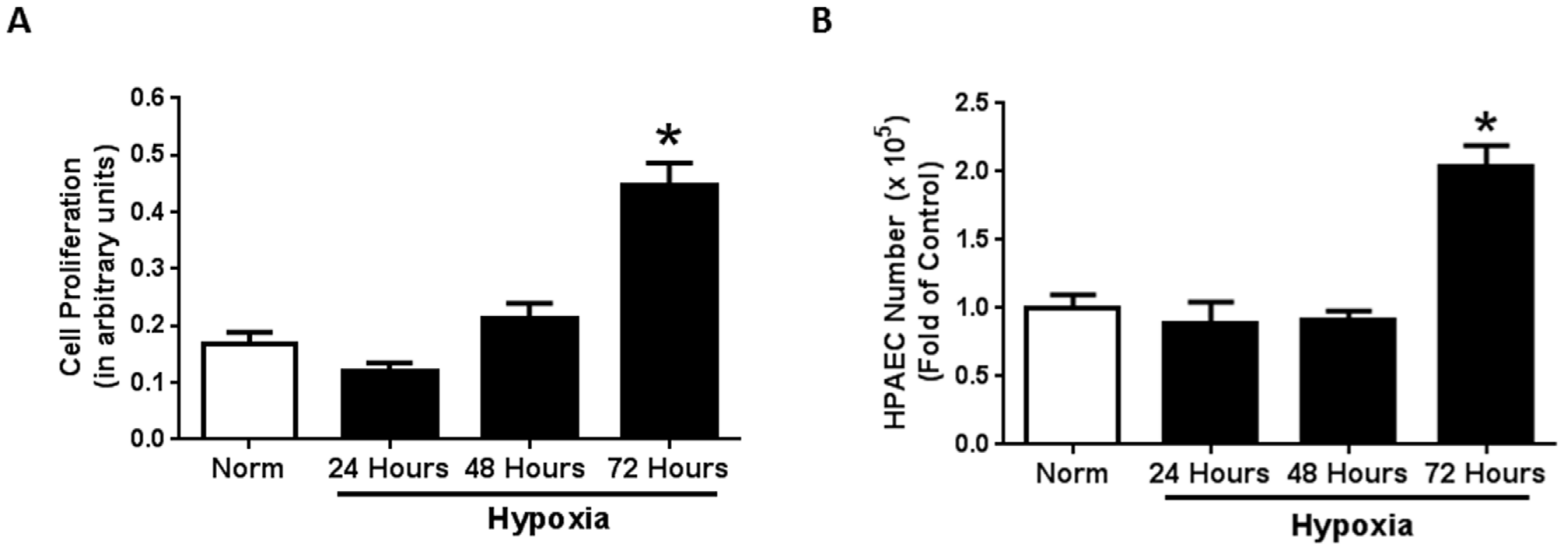
Prolonged hypoxia exposure increases endothelial cell proliferation. Seventy two hours of hypoxia exposure significantly stimulates endothelial cell proliferation when compared to all other groups. Human pulmonary artery endothelial cells (HPAEC) were exposed to normoxic or hypoxic (1% O_2_) conditions for 24-, 48-, or 72 hours (n = 4). Following exposure, cell proliferation was assessed by MTT (Figure 1A) assay and Trypan Blue Dye Exclusion Assay (Figure 1B). * p<0.0001.

Hypoxia is associated with significant endothelial alterations which are thought to contribute to PH development and progression [3], [20]. Pulmonary arteries [4] and lung sections of PH patients demonstrate abnormal endothelial cell growth [5], [6]. To determine whether hypoxia alters HPAEC function *in vitro*, we assessed HPAEC proliferation following 24, 48, and 72 hours of hypoxia exposure. While 24 and 48 hours of hypoxia exposure had no effect on cellular proliferation, 72 hours of hypoxia increases cellular proliferation when measured by MTT assay (**Figure 1A**) and Trypan Blue Dye Exclusion Assay (**Figure 1B**).

### Chronic Hypoxia Exposure Stimulates HPAEC 5-Lipoxygenase Expression

**Figure 2:**
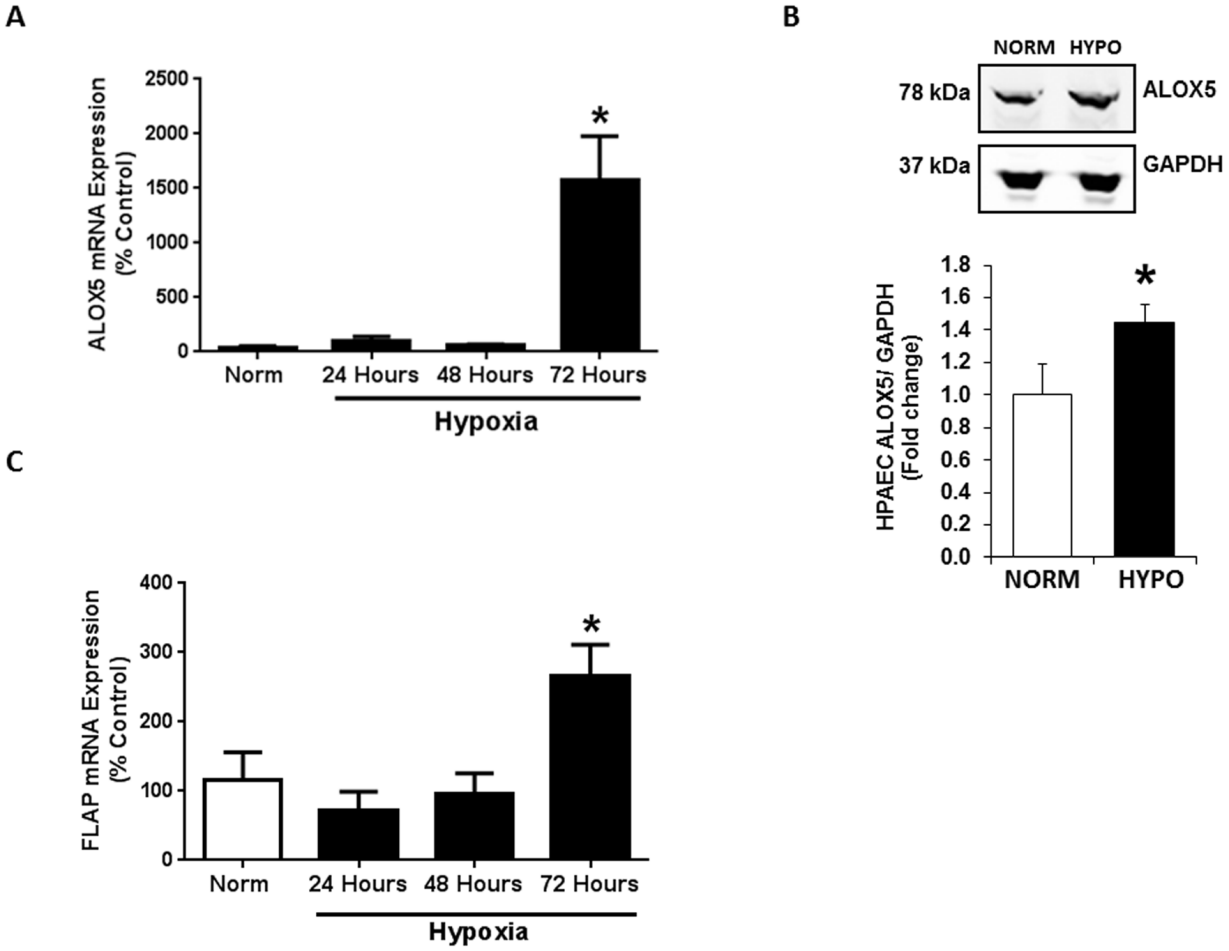
Chronic hypoxia exposure increases endothelial ALOX5 expression. Seventy two hours of hypoxia exposure significantly stimulates endothelial ALOX5 expression when compared to all other groups. HPAEC were exposed to normoxic or hypoxic conditions for 24-, 48-, or 72 hours. Following exposure, cells were collected, and total RNA and protein were isolated for expression analyses via quantitative real time PCR and Western blot respectively. Results indicate that ALOX5 mRNA levels are significantly increased following hypoxia exposure (A, n = 5). Chronic hypoxia exposure also causes a 3-fold elevation in ALOX5 protein expression levels (B, n = 4). Endothelial FLAP expression is also increased when compared to all other groups (C, n = 5–7). Values are expressed as percent of control. * p<0.001 when compared to all other groups.

*In vivo* studies demonstrate that both hypoxia exposure and MCT administration upregulate 5-lipoxygenase (ALOX5) [9], [21]. To specifically investigate the effect of hypoxia on pulmonary artery endothelial cell ALOX5, HPAEC were exposed to hypoxia for 24-, 48-, or 72-hours. Our results indicate that 72 hours of hypoxia exposure significantly increases HPAEC ALOX5 mRNA (**Figure 2A**) when analyzed by qRT-PCR and ALOX5 protein expression (**Figure 2B**) as measured by western blot. Prolonged hypoxia exposure also stimulates a significant increase in the expression of the ALOX5 required cofactor, FLAP when measured by qRT-PCR (**Figure 2C**).

### ALOX5 Gene Silencing Reduces Pulmonary Endothelial Proliferation Following Prolonged Hypoxia Exposure

**Figure 3:**
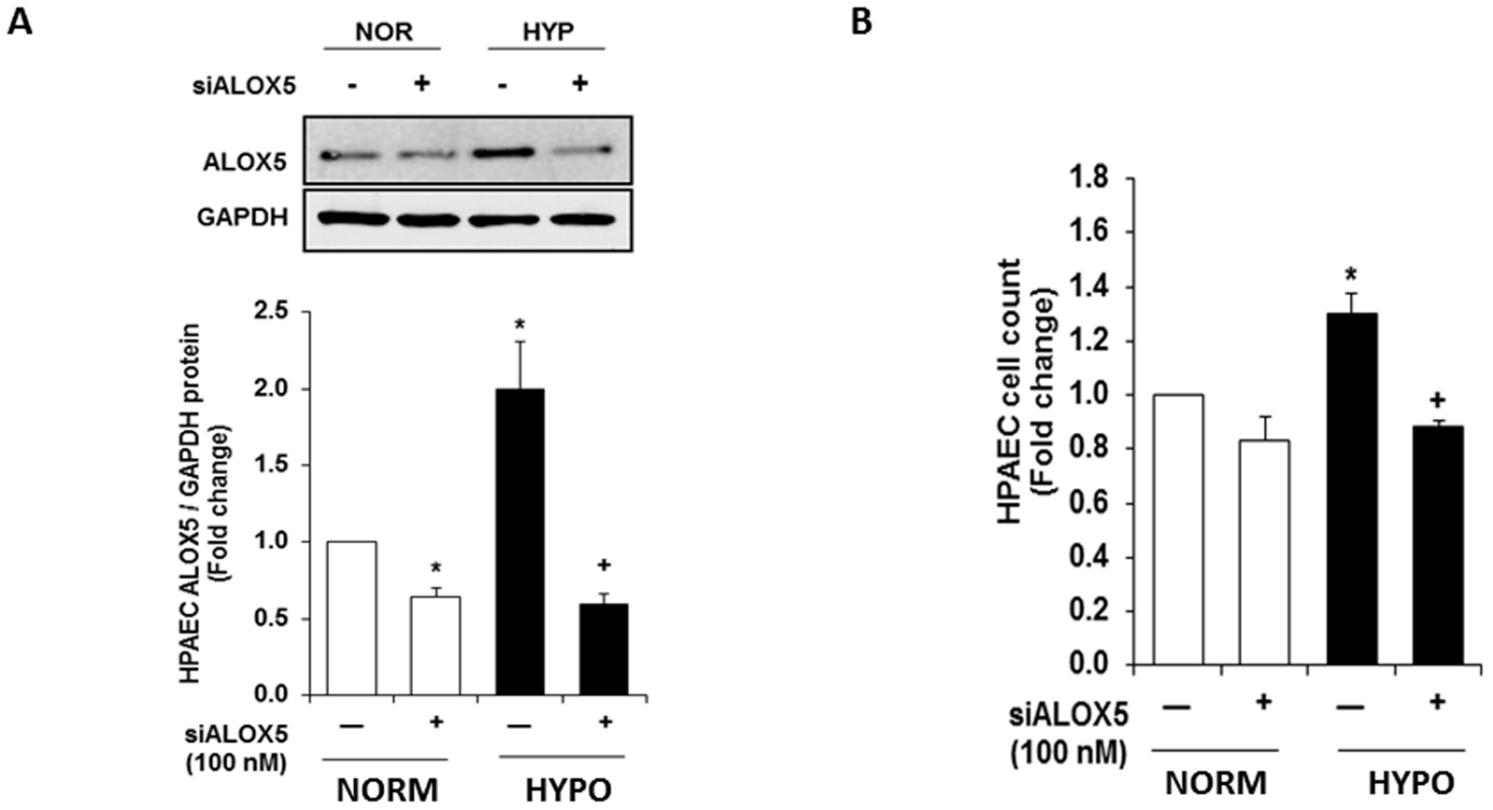
ALOX5 gene silencing prevents hypoxia-induced endothelial cell proliferation. HPAEC transfected with scrambled- and ALOX5-siRNA were exposed to normoxic or hypoxic conditions for 72 hours. Following exposure, cells were collected for ALOX5 expression analysis. Transfection with ALOX5 siRNA produces a 1.5 fold decrease in ALOX5 protein expression following hypoxia exposure (A, n = 5). Proliferation of control and transfected cells was also assessed following 72-hour exposure to normoxic and hypoxic conditions. ALOX5 gene silencing prevents endothelial cell proliferation during chronic hypoxia exposure (B, n = 5). Values are expressed as fold change. * p<0.01 when compared to normoxic groups, ** p<0.05 when compared to hypoxic controls.

Recent studies demonstrate that ALOX5 contributes to HPAEC proliferation [11]. Additionally, concomitant increases in ALOX5 and HPAEC proliferation following hypoxia exposure suggest a potential association between ALOX5 activity and hypoxia-induced endothelial proliferation. To determine whether ALOX5 mediates hypoxia-induced endothelial proliferation, human pulmonary artery endothelial cells (HPAEC) were transfected with control (scrambled) or ALOX5 silencing RNA. Transfection with ALOX5 siRNA reduces ALOX5 protein expression when compared to normoxic and hypoxic controls (**Figure 3A**). Densitometry reveals that ALOX5 siRNA transfection decreases hypoxia-induced ALOX5 protein levels by greater than 50% when compared to untreated hypoxic groups (**Figure 3A**). Following transfection, control- and siALOX5 human pulmonary artery endothelial cells were placed in normoxic or hypoxic conditions for 72 hours then collected for cell proliferation analyses. Transfection with ALOX5 siRNA significantly decreases hypoxia-induced HPAEC proliferation when compared to hypoxia scrambled-controls as measured by trypan blue dye exclusion assay (**Figure 3B**). These results indicate that ALOX5 is a significant contributor to hypoxia-induced increases in endothelial cell proliferation.

### Inhibition of ALOX5 Activity Attenuates Hypoxia-Induced Endothelial Proliferation

**Figure 4:**
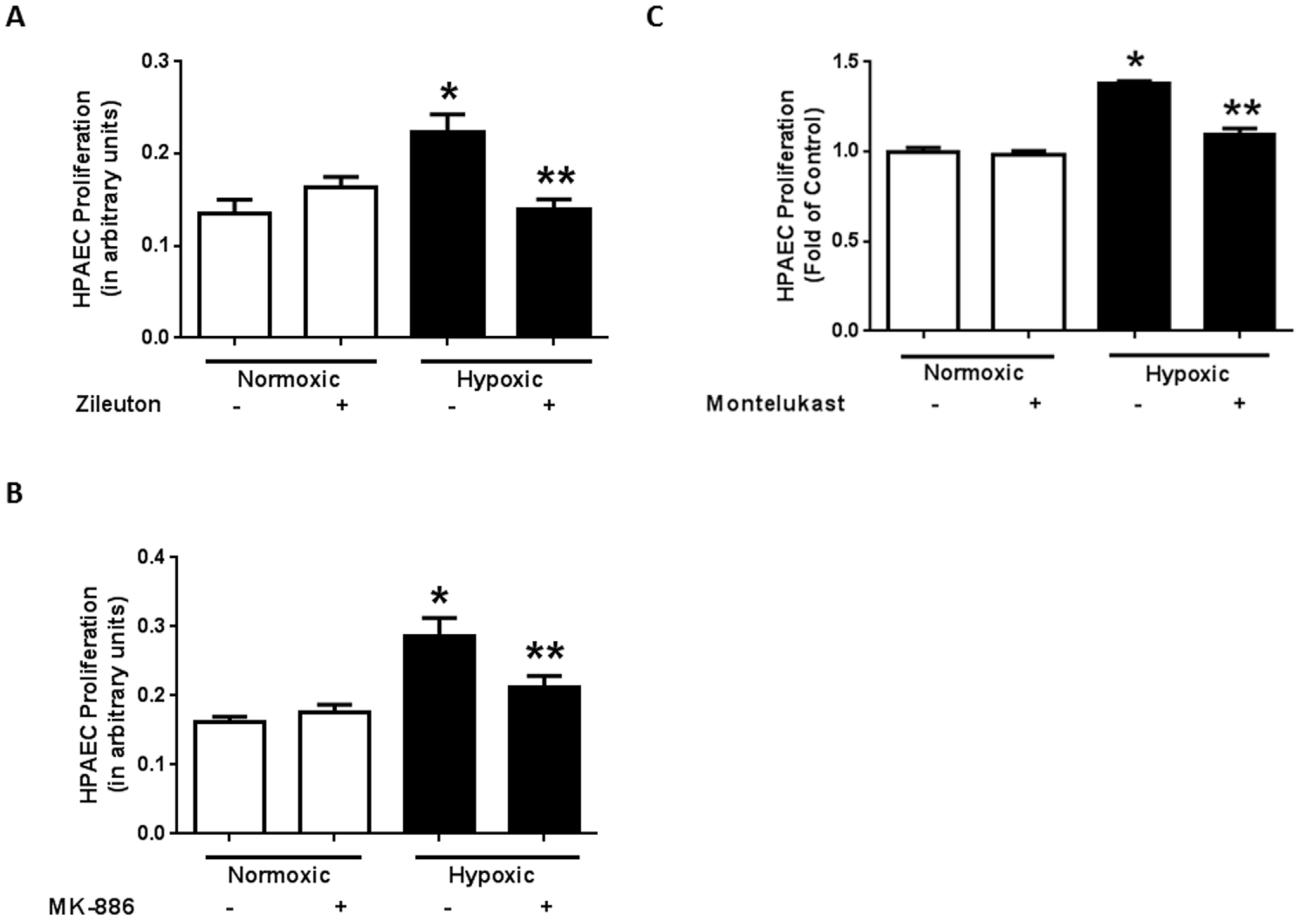
Pharmacological inhibition of ALOX5 signaling attenuates hypoxia-induced endothelial proliferation. ALOX5 blockade by zileuton administration reduces hypoxia-induced endothelial proliferation when measured by MTT Assay (A, n = 4). FLAP inhibition by MK-886 attenuates endothelial proliferation following hypoxia exposure (B, n = 4) Pre-treatment with the cysteinyl leukotriene receptor antagonist, montelukast prevents endothelial proliferation during prolonged hypoxia exposure (C, n = 6) HPAEC were exposed to normoxic or hypoxic conditions for 72 hours. ALOX5 inhibitors, zileuton (10 µM) and MK-886 (0.5 µM) were administered during the final 24 hours of normoxia or hypoxia exposure. Cell proliferation was then assessed by MTT assay. * p<0.05 when compared to normoxic groups. ** p<0.05 when compared to untreated hypoxic groups.

We next sought to determine whether alterations in ALOX5 activity contribute to hypoxia-induced endothelial proliferation. To confirm that ALOX5 promotes hypoxia-induced HPAEC proliferation, we measured cellular proliferation in response to hypoxia in the presence or absence of the ALOX5 inhibitor, zileuton. Inhibition of ALOX5 enzyme activity by zileuton administration during the final 24 hours of hypoxia exposure attenuates hypoxia-induced increases in endothelial proliferation (**Figure 4A**). Similarly, inhibition of the ALOX5 cofactor, FLAP by MK-886 reduces endothelial cell proliferation following hypoxia exposure (**Figure 4B**). Moreover, pre-treatment with the cysteinyl leukotriene receptor antagonist, montelukast prevents increases in endothelial proliferation during chronic hypoxia exposure (**Figure 4C**). These data demonstrate that ALOX5 and its LT metabolites mediate hypoxia-induced endothelial proliferation. Additionally, alterations in endothelial cell proliferation caused by hypoxia exposure are in part dependent on activation of CysLT receptors.

### Hypoxia Promotes Endothelial ROS Release

**Figure 5:**
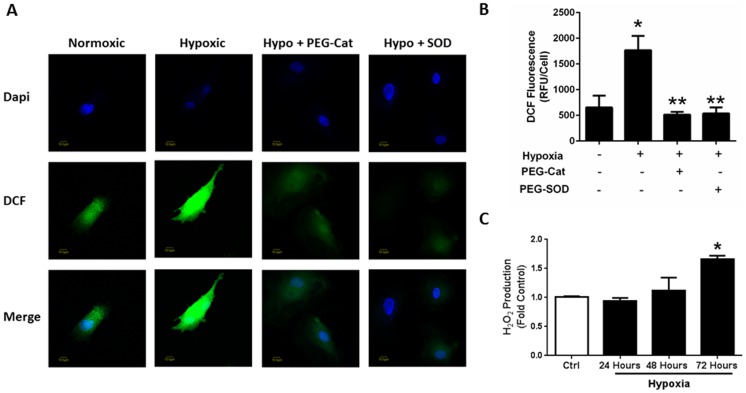
Hypoxia exposure stimulates endothelial ROS release. Human pulmonary artery endothelial cells were exposed to normoxic or hypoxic (1% O_2_) conditions for 24-, 48- or 72-hours. Following exposure, HPAEC ROS release was assessed by DCF staining (A, n = 3) and Amplex Red assay (C, n = 4). Results demonstrate that prolonged hypoxia exposure significantly increases endothelial ROS production whereas administration with the antioxidants, PEG- catalase or superoxide dismutase reduces these effects (B). Amplex Red Assay indicates that chronic hypoxia exposure promotes H_2_O_2_ release (C). * p<0.0001 when compared to normoxic controls.

Previous studies demonstrate that hypoxia promotes ROS generation in PH models [22]–[25]. To investigate whether hypoxia exposure increases endothelial ROS release, ROS release was analyzed following 24, 48, and 72-hours of hypoxia exposure. DCF staining indicates that chronic hypoxia exposure increases ROS production in endothelial cells (**Figure 5A**). This increase in ROS release was attenuated by the administration of PEG-catalase and superoxide dismutase (**Figure 5B**). Furthermore, Amplex Red assay analysis of hydrogen peroxide (H_2_O_2_) release also demonstrates that 72 hours of hypoxia exposure is required for increased in H_2_O_2_ (**Figure 5C**). These data suggest that chronic exposure to hypoxic conditions increases endothelial H_2_O_2_.

### H_2_O_2_ Mediates Hypoxia-Induced Increases in HPAEC ALOX5 Expression and Cell Proliferation

**Figure 6:**
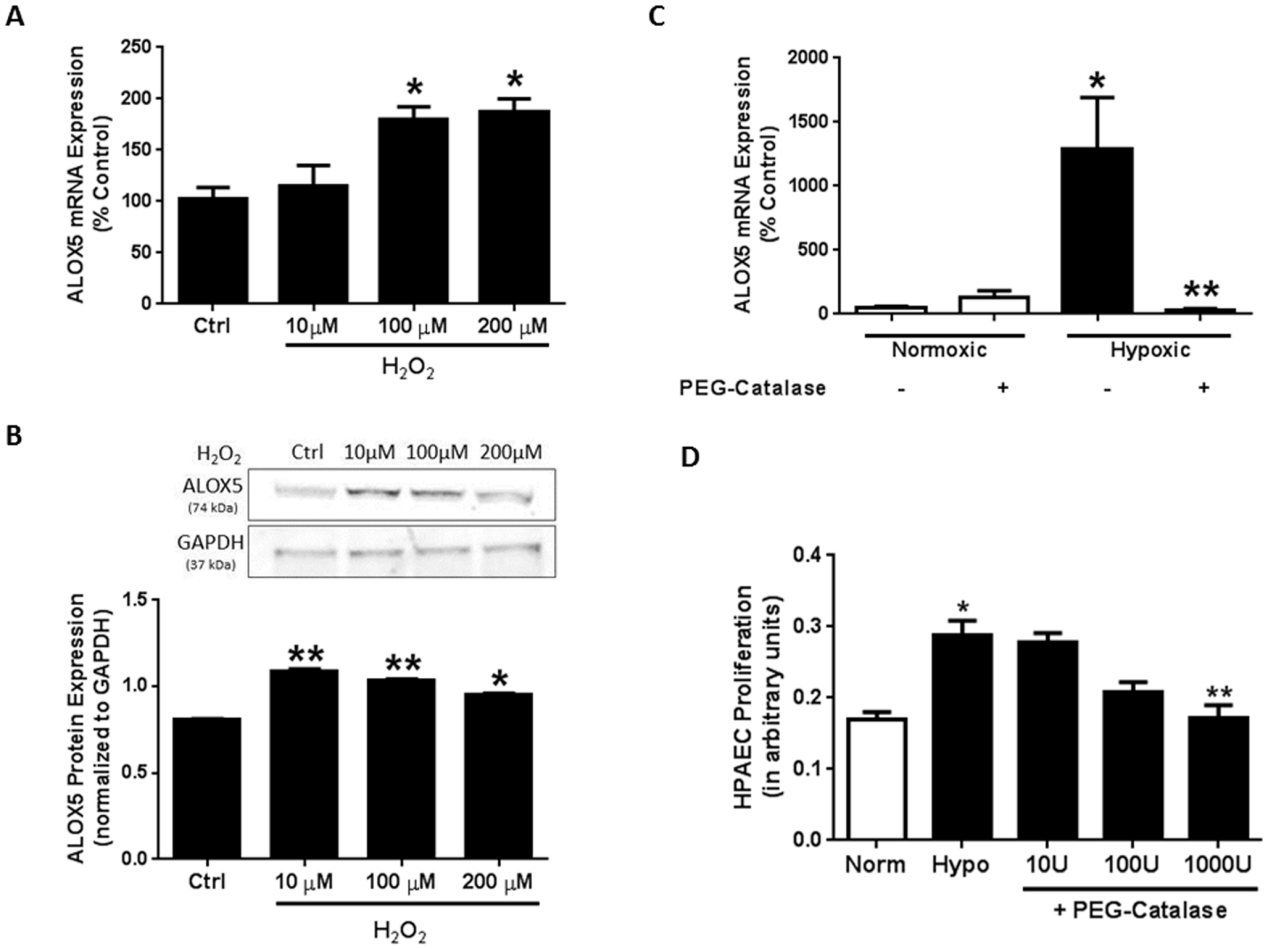
ROS mediate hypoxia-induced increases in endothelial ALOX5 expression and cell proliferation. Human pulmonary artery endothelial cells (HPAEC) were exposed to 0, 10, 100, and 200 µM hydrogen peroxide (H_2_O_2_) for 24 hours. Following exposure, supernatants were collected to assess cell toxicity by adenylate kinase release. Results demonstrate no significant changes in cell death as indicated by adenylate kinase release (n = 4–6; data not shown). HPAEC were collected and total RNA was isolated for quantitative real-time PCR gene expression analysis. ALOX5 was normalized to the housekeeping gene β-globin. Relative expression was calculated using the Delta-Delta C_T_ method and values were expressed as percent of control (A, n = 4–5). * p<0.05 when compared to untreated controls. H_2_O_2_ exposure stimulates HPAEC ALOX5 protein levels as analyzed by western blot (B, n = 4). PEG-Catalase (10U - 1000 U/ml) administration during the final 24 hours of the 72 hour hypoxia exposure prevents hypoxia-induced elevations in endothelial ALOX5 expression (C, n = 5) and cell proliferation (D, n = 6). * p<0.01 when compared to normoxic groups. ** p<0.05 when compared to untreated hypoxia controls.

Since elevated ROS production is associated with increased ALOX5 activity [14], [15], [17], we next sought to determine whether H_2_O_2_ mediates hypoxia-induced increases in endothelial ALOX5. HPAECs exposed to 0–200 µM concentrations of H_2_O_2_ for 24 hours revealed that 100 µM and 200 µM H_2_O_2_ concentrations increase endothelial ALOX5 mRNA levels (**Figure 6A**). Similarly, H_2_O_2_ exposure stimulates ALOX5 protein expression when assessed by Western blot (**Figure 6B**). These data indicate that ALOX5 expression levels are altered by increased ROS. To determine whether hypoxia-induced ROS mediate alterations in HPAEC ALOX5 levels and cell proliferation, we administered the antioxidant, PEG-catalase during the final 24 hours of the 72 hour hypoxia exposure period and determined ALOX5 mRNA levels by qRT-PCR and cell proliferation by MTT Assay. Results demonstrate that catalase administration significantly reduces hypoxia-induced elevations in endothelial ALOX5 gene expression (**Figure 6C**). Catalase treatment during the final 24 hours of the 72-hour hypoxia exposure also prevents HPAEC proliferation (**Figure 6D**) in a concentration-dependent manner when compared to untreated hypoxic groups.

## Discussion

Our studies indicate that hypoxia exposure promotes pulmonary artery endothelial proliferation by stimulating key catalytic molecules in the LT pathway, ALOX5 and its required co-factor, FLAP. We also demonstrate that hypoxia mediates these events by increasing ROS particularly H_2_O_2_. Overall, these studies suggest that ALOX5 contributes to hypoxia-induced endothelial proliferation in a ROS-dependent manner.

In this study, we demonstrate that prolonged hypoxia exposure significantly increases ALOX5 expression when compared to normoxic controls. Although ALOX5 was originally believed to be expressed only in myeloid cells, previous studies confirm ALOX5 expression in pulmonary artery endothelial cells [26]. Additionally, our data demonstrating hypoxia-induced elevations in HPAEC ALOX5 expression correspond with previous studies performed using rat lung homogenates and pulmonary arteries [27]. Furthermore, endothelial cells are shown to produce leukotrienes in nitric oxide-deficient and hypertensive conditions [28], [29]. These data demonstrate that endothelial cells express ALOX5 and are able to produce leukotrienes in environments of excessive oxidative stress and vascular injury. The results from our study provide similar evidence as pre-treatment with the cysteinyl LT receptor antagonist, montelukast prevents hypoxia-induced increases in HPAEC proliferation. Our data also suggest that endothelial ALOX5 expression is redox-sensitive as ALOX5 expression levels are increased in response to exogenous H_2_O_2_ administration (100 µM). Although the physiological concentrations of H_2_O_2_ are unknown, the concentrations used [30]–[32] are consistent with previous studies. Additionally, our results coincide with previous work indicating that ROS release stimulates ALOX5 [17].

This study also reveals that prolonged hypoxia exposure stimulates endothelial cell proliferation. Although previous studies indicate that hypoxia failed to stimulate endothelial cell proliferation [33], several studies demonstrate that hypoxia exposure produces significant elevations in endothelial cell proliferation [18], [34]. Though it is widely-accepted that hypoxia promotes pulmonary vascular cell proliferation, the underlying mechanism remains unclear. Endothelial cells contribute to hypoxic pulmonary remodeling by increasing the release of pro-proliferative mediators such as endothelin-1 and angiotensin II and reducing the release of anti-proliferative agents such as nitric oxide. Similarly, hypoxia stimulates cell proliferation by increasing the production of pro-proliferative stimuli from smooth muscle cells, platelets, fibroblasts, and endothelial cells [35]–[38]. Our data indicate that hypoxia increases endothelial cell proliferation by stimulating the ALOX5 pathway. Endothelial proliferation was reduced following the inhibition of the ALOX5 pathway by siRNA knockdown of ALOX5 as well as zileuton, MK-886, and montelukast administration. Zileuton inhibits ALOX5 by binding to the iron atom needed for catalytic function [39]. MK-886, however, inhibits leukotriene synthesis [40] by binding to the membrane-bound FLAP and preventing translocation of ALOX5 from the cytoplasm to the plasma membrane for activation. Furthermore, targeted reductions in ALOX5 using siRNA similarly inhibited hypoxia-induced proliferation. These data implicate a contributing role of leukotrienes in hypoxia-induced endothelial proliferation.

The results of the present study also indicate that ROS such as H_2_O_2_ contribute to the hypoxia-induced endothelial cell proliferation, as PEG-Catalase treatment prevents hypoxia-induced endothelial proliferation. Interestingly, our data demonstrate a prolonged exposure to hypoxia (72 hours) is needed to induce pulmonary endothelial cell proliferation, whereas 24- and 48- hours of hypoxia exposure do not significantly impact endothelial cell proliferation. It is plausible that accumulation of H_2_O_2_ is needed for the induction of cellular proliferation and our H_2_O_2_ measurements demonstrate a delay in hypoxia-induced increases in H_2_O_2_ (Figure 6C). Interestingly, bolus H_2_O_2_ administration promotes ALOX5 expression and HPAEC proliferation following only 24 hours. This discrepancy most likely results from the exogenous administration of H_2_O_2_, which surpasses the normal cellular processes necessary for H_2_O_2_ production during hypoxia exposure. Moreover, bolus H_2_O_2_ administration may also stimulate ALOX5 expression and HPAEC proliferation through the activation of downstream signaling pathways. Recent reports indicate that H_2_O_2_ activates endothelial p38 MAPK [41], [42] and NF-kappaB activity [43], [44], two mediators associated with increases in ALOX5 expression and activity. Furthermore, the addition of PEG-catalase is shown to double endogenous endothelial catalase activity within 4 hours [45]. Taken together, these findings are consistent with previous reports demonstrating that the highly reactive oxidant, peroxynitrite stimulates pulmonary artery endothelial cell proliferation [46] and catalase inhibits cell proliferation [47]. The mechanism responsible for an ALOX5-mediated increase in proliferation is unclear however, It is possible that ALOX5 promotes cell proliferation via its nuclear localization or through interaction with cytoskeletal proteins [48], [49]. Nonetheless, the excessive proliferation of pulmonary endothelial cells is thought to contribute to the obliteration of the pulmonary artery vessel lumen. Therefore, it is likely that hypoxia-induced pulmonary artery endothelial proliferation contributes to pulmonary hypertension (PH) development and progression.

Previous research demonstrates that chronic hypoxia significantly increases endothelial cell proliferation. *In vivo* studies indicate that endothelial cells in the main pulmonary artery and in the small muscular arteries are increased in chronically hypoxic rats [50], [51]. Endothelial proliferation is also increased in neonatal calves following exposure to 8% oxygen for 14 days [52]. Additionally, excessive endothelial proliferation leads to plexiform lesion formation in idiopathic PAH patients [53]. Our research, performed *in vitro*, similarly demonstrates that hypoxia promotes endothelial proliferation. We also show that hypoxia-induced endothelial proliferation is ROS-dependent as the administration of PEG-catalase attenuates these events. These hypoxia-induced ROS are likely produced by NADPH oxidases (Noxes) as previous studies indicate that Nox4 expression is elevated in low oxygen environments [25]. Interestingly, previous studies also suggest that NADPH oxidase activity is required for endothelial cell proliferation [54]. Additionally, studies by our group and others have implicated Nox4 in the hypoxia-induced proliferation of human pulmonary artery smooth muscle cells [19], [55]. Hypoxia-induced increases in ROS may also result from reductions in antioxidant availability, as hypoxia exposure significantly decreases glutathione levels in porcine pulmonary artery endothelial cells [56].

Although our evidence indicates that hypoxia increases ALOX5 in a redox-sensitive manner, the mechanisms underlying these events remain unclear. Two major pathways are implicated in regulating ALOX5 expression: promoter methylation and transcription factor activation [57]. Research demonstrates that leukocyte cell lines that have methylated promoters, U-937 and HL-60TB, do not express ALOX5, while the HL-60 cell line, which is unmethylated expresses ALOX5 [58], [59]. Moreover, the treatment of the U-937 and HL-60TB cell lines with the demethylating agent, 5-aza-2'deoxycytidine resulted in the restoration of ALOX5 expression [58]. There are also 5 GC boxes that bind SP1 and have been shown to be important in regulating expression of ALOX5 [60] however, ablation of all GC-boxes only reduces ALOX5 promoter activity 47%, implicating other transcription factors such as hypoxia- inducible factor (HIF) in ALOX5 regulation. The oxygen-sensing molecule, HIF-1α [61] regulates the adaptive response by activating genes associated with energy metabolism, erythropoiesis, vasomotor tone, and angiogenesis [62], and recent work demonstrates that HIF-1α mediates hypoxia-induced FLAP expression in human pulmonary microvascular endothelial cells [63]. HIF-2α also contributes to the vascular response to chronic hypoxia including the expression of genes involved in pulmonary vascular cell proliferation [64]. Hypoxia may also modulate ALOX5 via the early growth response protein-1, or Egr-1. This hypoxia-inducible transcription factor [65] has a binding site within the ALOX5 promoter region [66] and is expressed in a variety of pulmonary vascular cells, including endothelial cells [67]. Research also demonstrates that Egr-1 acts in a redox-sensitive manner, as Egr-1 is upregulated by ROS release and attenuated following antioxidant overexpression [68], [69]. In addition to HIF and Egr-1, the ALOX5 promoter region also contains binding sites for TGF-β [70] and NF-κB [71], [72], which are also implicated in the endothelial dysfunction [73], [74] and vascular remodeling [75] in PH pathogenesis [76], [77].

In summary, the current study demonstrates that hypoxia-induced ROS plays a critical role regulating ALOX5 and endothelial proliferation. Our data suggests that ALOX5 mediates hypoxia-induced endothelial cell proliferation and implicates increased ALOX5 expression and activity in vascular remodeling in experimental and clinical PH. Overall, these results suggest that ALOX5 inhibition merits additional investigation as a therapeutic target for the prevention or treatment of PH.

## References

[1] Wright JL, Lawson L, Pare PD, Hooper RO, Peretz DI, et al (1983) The structure and function of the pulmonary vasculature in mild chronic obstructive pulmonary disease. The effect of oxygen and exercise. Am Rev Respir Dis 128: 702–707. 6625346 10.1164/arrd.1983.128.4.702

[2] Orr R, Smith LJ, Cuttica MJ (2012) Pulmonary hypertension in advanced chronic obstructive pulmonary disease. Curr Opin Pulm Med 18: 138–143. 22189454 10.1097/MCP.0b013e32834f2093

[3] Tuder RM, Groves B, Badesch DB, Voelkel NF (1994) Exuberant endothelial cell growth and elements of inflammation are present in plexiform lesions of pulmonary hypertension. Am J Pathol 144: 275–285. 7508683 PMC1887146

[4] Sakao S, Tatsumi K, Voelkel N (2009) Endothelial cells and pulmonary arterial hypertension: apoptosis, proliferation, interaction and transdifferentiation. Respiratory Research 10: 95. 19825167 10.1186/1465-9921-10-95 PMC2768704

[5] Masri F, Xu W, Comhair S, Asosingh K, Koo M, et al (2007) Hyperproliferative apoptosis-resistant endothelial cells in idiopathic pulmonary arterial hypertension. Am J Physiol Lung Cell Mol Physiol 293: 548–554. 10.1152/ajplung.00428.2006 17526595

[6] Rabinovitch M, Bothwell T, Hayakawa BN, Williams WG, Trusler GA, et al (1986) Pulmonary artery endothelial abnormalities in patients with congenital heart defects and pulmonary hypertension. A correlation of light with scanning electron microscopy and transmission electron microscopy. Lab Invest 55: 632–653. 3784535

[7] Morganroth ML, Stenmark KR, Morris KG, Murphy RC, Mathias M, et al (1985) Diethylcarbamazine inhibits acute and chronic hypoxic pulmonary hypertension in awake rats. Am Rev Respir Dis 131: 488–492. 3994144 10.1164/arrd.1985.131.4.488

[8] Stenmark KR, Morganroth ML, Remigio LK, Voelkel NF, Murphy RC, et al (1985) Alveolar inflammation and arachidonate metabolism in monocrotaline-induced pulmonary hypertension. Am J Physiol 248: H859–866. 3923843 10.1152/ajpheart.1985.248.6.H859

[9] Jones JE, Walker JL, Song Y, Weiss N, Cardoso WV, et al (2004) Effect of 5-lipoxygenase on the development of pulmonary hypertension in rats. Am J Physiol Heart Circ Physiol 286: H1775–1784. 14726295 10.1152/ajpheart.00281.2003

[10] Friedman Z, Lunyong VE, Courtney J, Smith H, Berkowitz P, et al (1984) Prostaglandin formation in the isolated human ductus arteriosus, aorta, pulmonary and umbilical arteries. Prostaglandins Leukot Med 14: 279–286. 6429672 10.1016/0262-1746(84)90211-7

[11] Walker JL, Loscalzo J, Zhang YY (2002) 5-Lipoxygenase and human pulmonary artery endothelial cell proliferation. Am J Physiol Heart Circ Physiol 282: H585–593. 11788406 10.1152/ajpheart.00003.2001

[12] Miller DK, Gillard JW, Vickers PJ, Sadowski S, Leveille C, et al (1990) Identification and isolation of a membrane protein necessary for leukotriene production. Nature 343: 278–281. 2300172 10.1038/343278a0

[13] Dixon RA, Diehl RE, Opas E, Rands E, Vickers PJ, et al (1990) Requirement of a 5-lipoxygenase-activating protein for leukotriene synthesis. Nature 343: 282–284. 2300173 10.1038/343282a0

[14] Riendeau D, Denis D, Choo LY, Nathaniel DJ (1989) Stimulation of 5-lipoxygenase activity under conditions which promote lipid peroxidation. Biochem J 263: 565–572. 2512907 10.1042/bj2630565 PMC1133464

[15] Hatzelmann A, Ullrich V (1987) Regulation of 5-lipoxygenase activity by the glutathione status in human polymorphonuclear leukocytes. Eur J Biochem 169: 175–184. 2824200 10.1111/j.1432-1033.1987.tb13595.x

[16] Hatzelmann A, Schatz M, Ullrich V (1989) Involvement of glutathione peroxidase activity in the stimulation of 5-lipoxygenase activity by glutathione-depleting agents in human polymorphonuclear leukocytes. Eur J Biochem 180: 527–533. 2496978 10.1111/j.1432-1033.1989.tb14678.x

[17] Werz O, Szellas D, Steinhilber D (2000) Reactive oxygen species released from granulocytes stimulate 5-lipoxygenase activity in a B-lymphocytic cell line. Eur J Biochem 267: 1263–1269. 10691962 10.1046/j.1432-1327.2000.01000.x

[18] Kang B-Y, Kleinhenz JM, Murphy TC, Hart CM (2011) The PPAR-gamma ligand rosiglitazone attenuates hypoxia-induced endothelin signaling in vitro and in vivo. American Journal of Physiology - Lung Cellular and Molecular Physiology 301: L881–L891. 21926265 10.1152/ajplung.00195.2011 PMC3233829

[19] Green DE, Murphy TC, Kang BY, Kleinhenz JM, Szyndralewiez C, et al.. (2012) The Nox4 Inhibitor, GKT137831, Attenuates Hypoxia-Induced Pulmonary Vascular Cell Proliferation. Am J Respir Cell Mol Biol. 10.1165/rcmb.2011-0418OC PMC3547100 22904198

[20] Schaefer CA, Kuhlmann CR, Weiterer S, Fehsecke A, Abdallah Y, et al (2006) Statins inhibit hypoxia-induced endothelial proliferation by preventing calcium-induced ROS formation. Atherosclerosis 185: 290–296. 16112121 10.1016/j.atherosclerosis.2005.06.035

[21] Voelkel NF, Tuder RM, Wade K, Höper M, Lepley RA, et al (1996) Inhibition of 5-lipoxygenase-activating protein (FLAP) reduces pulmonary vascular reactivity and pulmonary hypertension in hypoxic rats. The Journal of Clinical Investigation 97: 2491–2498. 8647941 10.1172/JCI118696 PMC507334

[22] Fresquet F, Pourageaud F, Leblais V, Brandes RP, Savineau JP, et al (2006) Role of reactive oxygen species and gp91phox in endothelial dysfunction of pulmonary arteries induced by chronic hypoxia. Br J Pharmacol 148: 714–723. 16715116 10.1038/sj.bjp.0706779 PMC1751862

[23] Wang X, Tong M, Chinta S, Raj JU, Gao Y (2006) Hypoxia-induced reactive oxygen species downregulate ETB receptor-mediated contraction of rat pulmonary arteries. Am J Physiol Lung Cell Mol Physiol 290: L570–578. 16227321 10.1152/ajplung.00262.2005

[24] Weerackody RP, Welsh DJ, Wadsworth RM, Peacock AJ (2009) Inhibition of p38 MAPK reverses hypoxia-induced pulmonary artery endothelial dysfunction. Am J Physiol Heart Circ Physiol 296: H1312–1320. 19201999 10.1152/ajpheart.00977.2008 PMC2685327

[25] Nisbet RE, Bland JM, Kleinhenz DJ, Mitchell PO, Walp ER, et al (2009) Rosiglitazone Attenuates Chronic Hypoxia-Induced Pulmonary Hypertension in a Mouse Model. Am J Respir Cell Mol Biol 42: 482–490. 19520921 10.1165/rcmb.2008-0132OC PMC2848739

[26] Zhang Y-Y, Walker JL, Huang A, Keaney JF, Clish CB, et al (2002) Expression of 5-lipoxygenase in pulmonary artery endothelial cells. Biochem J 361: 267–276. 11772398 10.1042/0264-6021:3610267 PMC1222306

[27] Burke DL, Frid MG, Kunrath CL, Karoor V, Anwar A, et al (2009) Sustained hypoxia promotes the development of a pulmonary artery-specific chronic inflammatory microenvironment. American Journal of Physiology - Lung Cellular and Molecular Physiology 297: L238–L250. 19465514 10.1152/ajplung.90591.2008 PMC2742800

[28] Stanke-Labesque F, Devillier P, Veitl S, Caron F, Cracowski JL, et al (2001) Cysteinyl leukotrienes are involved in angiotensin II-induced contraction of aorta from spontaneously hypertensive rats. Cardiovasc Res 49: 152–160. 11121807 10.1016/s0008-6363(00)00238-8

[29] Stanke-Labesque F, Hardy G, Caron F, Cracowski JL, Bessard G (2003) Inhibition of leukotriene synthesis with MK-886 prevents a rise in blood pressure and reduces noradrenaline-evoked contraction in L-NAME-treated rats. Br J Pharmacol 140: 186–194. 12967948 10.1038/sj.bjp.0705405 PMC1574003

[30] Ishii Y, Partridge CA, Del Vecchio PJ, Malik AB (1992) Tumor necrosis factor-alpha-mediated decrease in glutathione increases the sensitivity of pulmonary vascular endothelial cells to H2O2. J Clin Invest 89: 794–802. 1541673 10.1172/JCI115658 PMC442924

[31] Breton-Romero R, Lamas S (2013) Hydrogen Peroxide Signaling Mediator in the Activation of p38 MAPK in Vascular Endothelial Cells. Methods Enzymol 528: 49–59. 23849858 10.1016/B978-0-12-405881-1.00003-3

[32] Jin BY, Lin AJ, Golan DE, Michel T (2012) MARCKS protein mediates hydrogen peroxide regulation of endothelial permeability. Proc Natl Acad Sci U S A 109: 14864–14869. 22927426 10.1073/pnas.1204974109 PMC3443126

[33] Yu L, Hales C (2011) Hypoxia does neither stimulate pulmonary artery endothelial cell proliferation in mice and rats with pulmonary hypertension and vascular remodeling nor in human pulmonary artery endothelial cells. J Vasc Res 48: 465–475. 21691120 10.1159/000327005 PMC3128130

[34] Toby IT, Chicoine LG, Cui H, Chen B, Nelin LD (2010) Hypoxia-induced proliferation of human pulmonary microvascular endothelial cells depends on epidermal growth factor receptor tyrosine kinase activation. Am J Physiol Lung Cell Mol Physiol 298: L600–606. 20139181 10.1152/ajplung.00122.2009 PMC2853344

[35] Kourembanas S, Hannan RL, Faller DV (1990) Oxygen tension regulates the expression of the platelet-derived growth factor-B chain gene in human endothelial cells. J Clin Invest 86: 670–674. 2384608 10.1172/JCI114759 PMC296775

[36] Kourembanas S, Marsden PA, McQuillan LP, Faller DV (1991) Hypoxia induces endothelin gene expression and secretion in cultured human endothelium. J Clin Invest 88: 1054–1057. 1885767 10.1172/JCI115367 PMC295521

[37] Humar R, Kiefer FN, Berns H, Resink TJ, Battegay EJ (2002) Hypoxia enhances vascular cell proliferation and angiogenesis in vitro via rapamycin (mTOR)-dependent signaling. FASEB J 16: 771–780. 12039858 10.1096/fj.01-0658com

[38] Mukhopadhyay D, Tsiokas L, Zhou XM, Foster D, Brugge JS, et al (1995) Hypoxic induction of human vascular endothelial growth factor expression through c-Src activation. Nature 375: 577–581. 7540725 10.1038/375577a0

[39] Carter GW, Young PR, Albert DH, Bouska J, Dyer R, et al (1991) 5-lipoxygenase inhibitory activity of zileuton. J Pharmacol Exp Ther 256: 929–937. 1848634

[40] Rouzer CA, Ford-Hutchinson AW, Morton HE, Gillard JW (1990) MK886, a potent and specific leukotriene biosynthesis inhibitor blocks and reverses the membrane association of 5-lipoxygenase in ionophore-challenged leukocytes. J Biol Chem 265: 1436–1442. 2104841

[41] Breton-Romero R, Gonzalez de Orduna C, Romero N, Sanchez-Gomez FJ, de Alvaro C, et al (2012) Critical role of hydrogen peroxide signaling in the sequential activation of p38 MAPK and eNOS in laminar shear stress. Free Radic Biol Med 52: 1093–1100. 22281399 10.1016/j.freeradbiomed.2011.12.026

[42] Usatyuk PV, Vepa S, Watkins T, He D, Parinandi NL, et al (2003) Redox regulation of reactive oxygen species-induced p38 MAP kinase activation and barrier dysfunction in lung microvascular endothelial cells. Antioxid Redox Signal 5: 723–730. 14588145 10.1089/152308603770380025

[43] Lee YJ, Kang IJ, Bunger R, Kang YH (2004) Enhanced survival effect of pyruvate correlates MAPK and NF-kappaB activation in hydrogen peroxide-treated human endothelial cells. J Appl Physiol (1985) 96: 793–801 discussion 792. 14578369 10.1152/japplphysiol.00797.2003

[44] Ungvari Z, Orosz Z, Labinskyy N, Rivera A, Xiangmin Z, et al (2007) Increased mitochondrial H2O2 production promotes endothelial NF-kappaB activation in aged rat arteries. Am J Physiol Heart Circ Physiol 293: H37–47. 17416599 10.1152/ajpheart.01346.2006

[45] Beckman JS, Minor RL Jr, White CW, Repine JE, Rosen GM, et al (1988) Superoxide dismutase and catalase conjugated to polyethylene glycol increases endothelial enzyme activity and oxidant resistance. J Biol Chem 263: 6884–6892. 3129432

[46] Agbani EO, Coats P, Mills A, Wadsworth RM (2011) Peroxynitrite stimulates pulmonary artery endothelial and smooth muscle cell proliferation: involvement of ERK and PKC. Pulm Pharmacol Ther 24: 100–109. 20851205 10.1016/j.pupt.2010.09.003

[47] Zanetti M, Katusic ZS, O'Brien T (2002) Adenoviral-mediated overexpression of catalase inhibits endothelial cell proliferation. Am J Physiol Heart Circ Physiol 283: H2620–2626. 12427601 10.1152/ajpheart.00358.2001

[48] Provost P, Doucet J, Hammarberg T, Gerisch G, Samuelsson B, et al (2001) 5-Lipoxygenase interacts with coactosin-like protein. J Biol Chem 276: 16520–16527. 11297527 10.1074/jbc.M011205200

[49] Lepley RA, Fitzpatrick FA (1994) 5-Lipoxygenase contains a functional Src homology 3-binding motif that interacts with the Src homology 3 domain of Grb2 and cytoskeletal proteins. J Biol Chem 269: 24163–24168. 7929073

[50] Howell K, Preston RJ, McLoughlin P (2003) Chronic hypoxia causes angiogenesis in addition to remodelling in the adult rat pulmonary circulation. J Physiol 547: 133–145. 12562951 10.1113/jphysiol.2002.030676 PMC2342608

[51] Meyrick B, Reid L (1979) Hypoxia and incorporation of 3H-thymidine by cells of the rat pulmonary arteries and alveolar wall. Am J Pathol 96: 51–70. 464026 PMC2042349

[52] Stiebellehner L, Belknap JK, Ensley B, Tucker A, Orton EC, et al (1998) Lung endothelial cell proliferation in normal and pulmonary hypertensive neonatal calves. Am J Physiol 275: L593–600. 9728055 10.1152/ajplung.1998.275.3.L593

[53] Voelkel NF, Tuder RM (1997) Cellular and molecular biology of vascular smooth muscle cells in pulmonary hypertension. Pulm Pharmacol Ther 10: 231–241. 9778486 10.1006/pupt.1998.0100

[54] Abid MR, Kachra Z, Spokes KC, Aird WC (2000) NADPH oxidase activity is required for endothelial cell proliferation and migration. FEBS Lett 486: 252–256. 11119713 10.1016/s0014-5793(00)02305-x

[55] Ismail S, Sturrock A, Wu P, Cahill B, Norman K, et al (2009) NOX4 mediates hypoxia-induced proliferation of human pulmonary artery smooth muscle cells: the role of autocrine production of transforming growth factor-{beta}1 and insulin-like growth factor binding protein-3. Am J Physiol Lung Cell Mol Physiol 296: L489–499. 19036873 10.1152/ajplung.90488.2008 PMC2660216

[56] Bhat GB, Tinsley SB, Tolson JK, Patel JM, Block ER (1992) Hypoxia increases the susceptibility of pulmonary artery endothelial cells to hydrogen peroxide injury. J Cell Physiol 151: 228–238. 1572899 10.1002/jcp.1041510203

[57] Radmark O, Samuelsson B (2005) Regulation of 5-lipoxygenase enzyme activity. Biochem Biophys Res Commun 338: 102–110. 16122704 10.1016/j.bbrc.2005.08.013

[58] Uhl J, Klan N, Rose M, Entian KD, Werz O, et al (2002) The 5-lipoxygenase promoter is regulated by DNA methylation. J Biol Chem 277: 4374–4379. 11706027 10.1074/jbc.M107665200

[59] Katryniok C, Schnur N, Gillis A, von Knethen A, Sorg BL, et al (2010) Role of DNA methylation and methyl-DNA binding proteins in the repression of 5-lipoxygenase promoter activity. Biochim Biophys Acta 1801: 49–57. 19781662 10.1016/j.bbalip.2009.09.003

[60] Dishart D, Schnur N, Klan N, Werz O, Steinhilber D, et al (2005) GC-rich sequences in the 5-lipoxygenase gene promoter are required for expression in Mono Mac 6 cells, characterization of a novel Sp1 binding site. Biochim Biophys Acta 1738: 37–47. 16413224 10.1016/j.bbalip.2005.11.008

[61] Semenza GL (2007) Life with oxygen. Science 318: 62–64. 17916722 10.1126/science.1147949

[62] Semenza GL (2003) Targeting HIF-1 for cancer therapy. Nat Rev Cancer 3: 721–732. 13130303 10.1038/nrc1187

[63] Gonsalves CS, Kalra VK (2010) Hypoxia-mediated expression of 5-lipoxygenase-activating protein involves HIF-1alpha and NF-kappaB and microRNAs 135a and 199a-5p. J Immunol 184: 3878–3888. 20194722 10.4049/jimmunol.0902594

[64] Tuder RM, Flook BE, Voelkel NF (1995) Increased gene expression for VEGF and the VEGF receptors KDR/Flk and Flt in lungs exposed to acute or to chronic hypoxia. Modulation of gene expression by nitric oxide. J Clin Invest 95: 1798–1807. 7706486 10.1172/JCI117858 PMC295709

[65] Banks MF, Gerasimovskaya EV, Tucker DA, Frid MG, Carpenter TC, et al (2005) Egr-1 antisense oligonucleotides inhibit hypoxia-induced proliferation of pulmonary artery adventitial fibroblasts. J Appl Physiol 98: 732–738. 15475598 10.1152/japplphysiol.00821.2004

[66] Silverman ES, Le L, Baron RM, Hallock A, Hjoberg J, et al (2002) Cloning and functional analysis of the mouse 5-lipoxygenase promoter. Am J Respir Cell Mol Biol 26: 475–483. 11919084 10.1165/ajrcmb.26.4.4747

[67] Fu M, Zhu X, Zhang J, Liang J, Lin Y, et al (2003) Egr-1 target genes in human endothelial cells identified by microarray analysis. Gene 315: 33–41. 14557062 10.1016/s0378-1119(03)00730-3

[68] Nozik-Grayck E, Suliman HB, Majka S, Albietz J, Van Rheen Z, et al (2008) Lung EC-SOD overexpression attenuates hypoxic induction of Egr-1 and chronic hypoxic pulmonary vascular remodeling. Am J Physiol Lung Cell Mol Physiol 295: L422–430. 18599502 10.1152/ajplung.90293.2008 PMC2536799

[69] Hartney T, Birari R, Venkataraman S, Villegas L, Martinez M, et al (2011) Xanthine oxidase-derived ROS upregulate Egr-1 via ERK1/2 in PA smooth muscle cells; model to test impact of extracellular ROS in chronic hypoxia. PLoS One 6: e27531. 22140445 10.1371/journal.pone.0027531 PMC3225357

[70] Sorg BL, Klan N, Seuter S, Dishart D, Radmark O, et al (2006) Analysis of the 5-lipoxygenase promoter and characterization of a vitamin D receptor binding site. Biochim Biophys Acta 1761: 686–697. 16750418 10.1016/j.bbalip.2006.04.005

[71] Samuelsson B, Hoshiko S, Radmark O (1991) Characterization of the promoter of the human 5-lipoxygenase gene. Adv Prostaglandin Thromboxane Leukot Res 21A: 1–8. 1825525

[72] Hoshiko S, Radmark O, Samuelsson B (1990) Characterization of the human 5-lipoxygenase gene promoter. Proc Natl Acad Sci U S A 87: 9073–9077. 2251250 10.1073/pnas.87.23.9073 PMC55106

[73] Birukova AA, Adyshev D, Gorshkov B, Birukov KG, Verin AD (2005) ALK5 and Smad4 are involved in TGF-beta1-induced pulmonary endothelial permeability. FEBS Lett 579: 4031–4037. 16004987 10.1016/j.febslet.2005.06.018

[74] Lu Q, Harrington EO, Jackson H, Morin N, Shannon C, et al (2006) Transforming growth factor-beta1-induced endothelial barrier dysfunction involves Smad2-dependent p38 activation and subsequent RhoA activation. J Appl Physiol 101: 375–384. 16645187 10.1152/japplphysiol.01515.2005

[75] Botney MD, Bahadori L, Gold LI (1994) Vascular remodeling in primary pulmonary hypertension. Potential role for transforming growth factor-beta. Am J Pathol 144: 286–295. 8311113 PMC1887154

[76] Richter A, Yeager ME, Zaiman A, Cool CD, Voelkel NF, et al (2004) Impaired transforming growth factor-beta signaling in idiopathic pulmonary arterial hypertension. Am J Respir Crit Care Med 170: 1340–1348. 15361368 10.1164/rccm.200311-1602OC

[77] Harrison RE, Berger R, Haworth SG, Tulloh R, Mache CJ, et al (2005) Transforming growth factor-beta receptor mutations and pulmonary arterial hypertension in childhood. Circulation 111: 435–441. 15687131 10.1161/01.CIR.0000153798.78540.87

